# Demographics of Feline Lymphoma in Australian Cat Populations: 1705 Cases

**DOI:** 10.3390/vetsci11120641

**Published:** 2024-12-11

**Authors:** Peter Bennett, Peter Williamson, Rosanne Taylor

**Affiliations:** 1 Melbourne Veterinary School, Faculty of Science, The University of Melbourne, Parkville, VIC 3010, Australia; 2 The Sydney School of Veterinary Science, Faculty of Science, The University of Sydney, Camperdown, NSW 2006, Australia; p.williamson@sydney.edu.au (P.W.); rosanne.taylor@sydney.edu.au (R.T.)

**Keywords:** cat, feline, cancer, lymphoma, epidemiology, breed risk

## Abstract

Lymphoma is the most common haematopoietic cancer in cats with few large studies evaluating breed and sex as risk factors for the disease. Australia’s geographic isolation and quarantine rules have led to a potentially restricted genetic pool and, currently, there have not been any large local epidemiological studies reported. A total of 1705 lymphoma cases were identified from several sources and compared to a reference population of 85,741 cats, and represent cats that are presented to veterinary clinics. Odds ratios were calculated for each breed that included lymphoma cases, as well as sex, retroviral status, and immunophenotype. The distributions of age and weight in the lymphoma and control populations and proportions of lymphoma cases in anatomic locations were compared. Eight breeds were identified as displaying increased potential risk of lymphoma and three at decreased risk. Male cats were found to be at increased risk (OR 1.2, 95%CI: 1.1 to 1.3, *p* = 0.002). The lymphoma cases were older, with a median age of 11.7 years compared to 9.0 years (*p* < 0.0001), and weighed less, with a median weight of 3.7 kg compared to 4.0 kg (*p* = 0.010), than the control population. Several breeds were found to have significant variations in the proportions of anatomical presentations including the Siamese, Burmilla, Australian mist, ragdoll, British shorthair, and domestic cats. These findings require confirmation in future studies that address the limitations of this study, as outlined in the discussion.

## Simple Summary

Lymphoma is a common cancer in cats with a global distribution. Australia is geographically isolated with strict quarantine rules, limiting the importation of cats that have the potential to alter the genetics of the cat population, which in turn, could alter the breed and sex risk of lymphoma. The few studies that have evaluated breed and sex risk in Australia have been conducted in a small number of cats. This study has evaluated these factors in a large group of Australian cats, identifying eight breeds with potentially increased and three breeds with potentially decreased lymphoma risk. Male cats were at increased risk and six breeds were shown to have a higher proportion of lymphoma involving specific body locations. The limitations of this study require confirmation of the findings in future studies.

## 1. Introduction

Lymphoma is the most common haematopoietic cancer in cats with age-adjusted incidence rates of 160/100,000 cats at risk reported [1].

Previous studies evaluating breed risk or prevalence of lymphoma have, for the most part, been retrospective studies from University Teaching Hospitals or referral practises with populations from geographically limited areas. These sources of cases and controls might reflect the disease in the general feline population [2]. Identifying and obtaining ideal reference populations for studies that include cases from multiple sources is challenging. Estimates of the occurrence of disease from imperfect databases are used by field epidemiologists, noting that there is valuable information attained despite the limitations introduced [2]. Potential biases that could arise include the demographics of owners of purebred cats and their willingness to accept referral to specialist and university hospitals.

The majority of lymphoma studies in cats are relatively small, including less than 120 cases [3]. There are three exceptions: A study from Switzerland reported 2868 cases against a reference population derived from cats that had samples submitted to three pathology laboratories [4]. The second included 546 cases seen at the Veterinary Medical Teaching Hospital at the University of California, Davis, focusing on the disease in the post-FeLV period, including only 477 retrovirus negative cases further evaluated [5]. The last study was from the United Kingdom, including 271 lymphoma cases from a database derived from primary care practises [6].

Our study aimed to evaluate a large lymphoma patient population derived from a range of sources and compared to a potentially relevant Australian control population. These populations were evaluated for the influence of breed, sex, age, weight, retroviral status, and immunophenotype on lymphoma presentation.

## 2. Materials and Methods

Patient populations: Patient records (January 2000 to December 2022) from two referral centres, the University of Sydney Veterinary Teaching Hospital (UVTHS) and Melbourne Veterinary Specialist Centre (MVSC), were searched for the terms: lymphoma or lymphosarcoma. These were reviewed by a board-certified veterinary oncologist (P.B.) and cases were selected with a diagnosis of lymphoma based on appropriate clinical findings along with either cytological or histological confirmation. Data collected were the anatomical presentation, immunophenotype, retrovirus status, sex, neuter status, weight, and age. Anatomical sites with low numbers of cases (*n* < 15) were grouped together and called ‘Other’ during data analyses. This group included bladder, chronic lymphocytic leukaemia, laryngeal, musculoskeletal, ocular, oral, orbital, periarticular, peripheral nervous system, pulmonary, splenic, tonsillar, and tracheal locations. Pathology reports were available for review, as above, from an Australian-wide commercial veterinary reference laboratory (Idexx Laboratories, Rydalmere, NSW, Australia), between February 2012 and May 2015, that had been screened for the word lymphoma. A predominately general practice corporate group (Greencross Vets, Woolloongabba, QLD, Australia) searched their medical records from January 2012 to December 2015 for the word lymphoma. Complete medical records were reviewed as above. Anonymised data were recorded in a Microsoft Excel v2410 spreadsheet.

Reference populations: All feline patients from the databases of the two referral hospitals, UVTHS and MVSC, were extracted and the lymphoma cases removed. The breed, age, sex, weight at the last visit, and neuter status were recorded. The cats registered in six local council regions in 2015 from the Sydney and Melbourne regions were used as a more general representation of the Australian cat populations. The data retrieved included breed, sex, neuter status, and age when available. Population data from the reference laboratory and the corporate practice group was sought but was not available.

Analysis of results: The data were first analysed as a whole, and then the combined referral hospital data (UVTHS and MVSC) and the data from the other sites (Idexx, Greencross, council registration) were analysed separately. The breed, sex, neuter status, age, and weight were compared between the lymphoma and control populations.

Statistical Analysis:Analyses were performed using IBM SPSS v28.0.0 or Minitab v21.4. All reported breeds within the above populations were included. Odds ratios (ORs) with 95% Confidence Intervals (CI) for breed, sex, retroviral status, and immunophenotype were calculated using Pearson Chi-Square tests with comparisons made between the cases and the control population for each of these variables. The ORs were only calculated for breeds in which more than one case of lymphoma occurred. The Shapiro–Wilks test was used to test the continuous results for normal distribution. For data confirmed to have a normal distribution mean and standard error are reported, and if not normally distributed, median and 95% confidence interval (95% CI) or interquartile range (IQR) are reported. Comparisons of proportions were tested using the Pearson Chi-Square test. For continuous data that did not have a normal distribution, the Mann–Whitney U test was used. The Bonferroni correction was used to adjust for multiple comparisons. Statistical significance was set at a *p* value of <0.5.

## 3. Results

There were 1705 cases of lymphoma, and 85,741 controls extracted from the search data. Lymphoma cases included 769 cases from the referral hospitals, 156 cases from the corporate general practices, and 780 cases from the reference laboratory. The controls included 54,198 cats from council registrations and 31,543 from the referral hospitals. The breeds and ages of the cats in both groups were predominantly reported by the owners. Staff reviewed the information on populations in the hospitals, but there was no similar check for council registrations. A complete list of the numbers of lymphoma cases and controls by source is included in the Supplementary Materials (Table S1)

The lymphoma population included 933 males (55.7%) and 750 females (44.0%). Neuter status was not recorded for 319 (19%) cats, while 22 (1.3%) lacked any sex identification. The reference laboratory recorded 300 (38.4%) cats as not being neutered, compared to 2600 (3%) cats at the other lymphoma and control data sources combined. Neuter status was significantly more likely to be recorded in cases than controls (*p* < 0.0001), so this parameter was not evaluated further. There were 440 purebred cats, representing 32 breeds, with 18 cats being identified as purebred cross, and 1247 as domestic cats. The coat length of the domestic cats was not consistently included so all were combined into a single group. The age and weight of the cats did not conform to a normal distribution. The ages ranged from 0.3 to 21.4 years, with a median of 11.7 years (IQR: 8.7–14.0 years). The weights of the cats ranged from 2.1 to 9.7 kg, with a median of 4.7 kg (IQR: 3.5–5.2 kg). The lymphoma characteristics are listed in Table 1 with full details in the Supplementary Materials (Table S2).

The control population included 23,640 males (51.7%) and 22,101 females (48.3%) where sex was known. There were 40,000 cats without their sex recorded, predominantly in the council registration data. Among the studied cats with their sex recorded, the neuter status was not recorded for 12,196 cats (26.1%) and those not neutered varied from 2.5% in the council data to 19.4% at UVTHS. There were 21,167 (24.7%) cats recorded as purebred, which consisted of 52 breeds. There were 2383 (2.8%) cats recorded as being purebred crosses and 61,654 (71.9%) as domestic cats. The ages of the controls ranged from 0.1 to 27.5 years with a median of 9.0 years (IQR: 2.6–12.9 years). The weights ranged from 0.14 to 13.5 kg, with a median of 4.0 kg (IQR: 3.6–5.4 kg). Retrovirus results were not available for the control populations.

**Figure 1:**
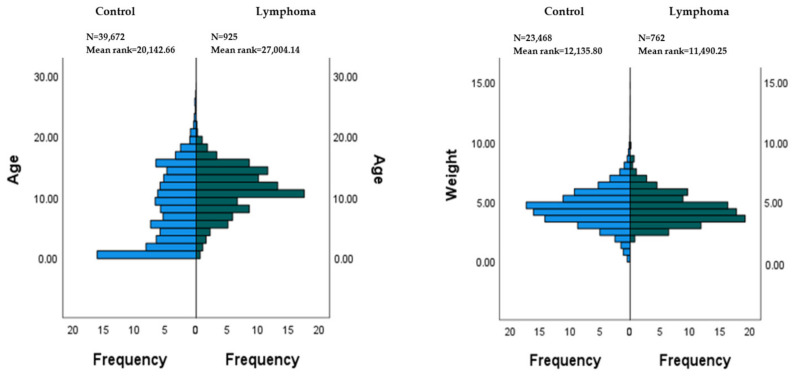
Age (years) and weight (kg) distributions of the control and lymphoma populations. Frequency values are percentages of the total for each age or weight.

Comparisons of the age and weight between the control and lymphoma populations showed different distributions with *p* values of 0.0001 for age and 0.012 for weight, respectively, with lymphoma cases being older and lighter. These results are illustrated in Figure 1.

**Figure 2:**
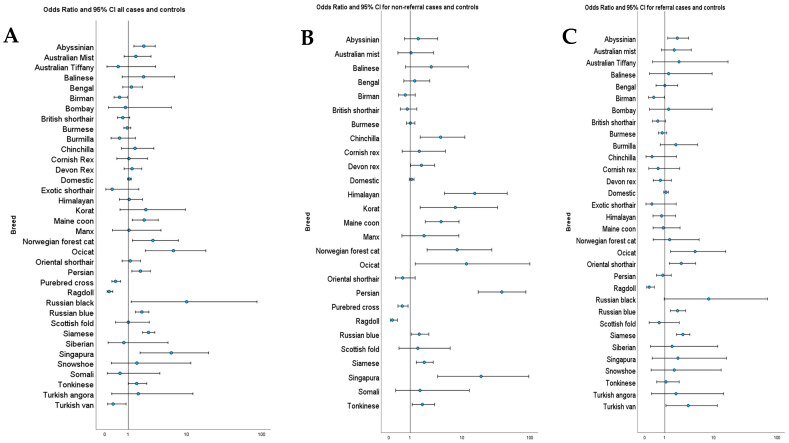
Odds ratios for lymphoma with 95% CI for all breeds with cases. (**A**) All cases and controls, (**B**) cases and controls from referral centres, and (**C**) cases and controls from non-referral centres. Circles represent the OR and the line is the 95% CI. The X axis scale is logarithmic.

Odds ratios were calculated for each breed that had more than one case of lymphoma. There were eight breeds at increased risk of lymphoma and three at decreased risk of lymphoma, along with pure breed crosses. The results are listed in Table 2. The ORs and 95% CI for all breeds that had cases of lymphoma, including the overall, referral only, and general populations, are illustrated in Figure 2. The full list of odds ratios for all breeds is included in the Supplementary Materials (Table S3).

When the ORs were calculated using only the referral populations, five breeds retained statistically significant increased risk, and two retained decreased risk. Two breeds lost statistically significant increased risk, and the Turkish van changed from a significantly decreased risk to an increased risk (OR 3.5, 95% CI: 1.1 to 11.5, *p* = 0.004). When the non-referral populations were used, seven breeds retained statistically significant increased risk, five breeds gained increased risk, two retained decreased risk, and two breeds were no longer identified as being at increased risk. Full details are included in the Supplementary Materials (Table S3).

Breed proportions varied between the groups from different sources. Domestic cats constituted a higher proportion in both the control (*p* < 0.001) and lymphoma (*p* = 0.011) groups of the non-referral population than in the referral population. The control populations included 27 breeds with a higher proportion in the referral than the general population. There were seven breeds with a higher proportion in the non-referral population controls than in the referral data. In the lymphoma cases, there were proportionally more Abyssinian (*p* = 0.018), Burmilla (*p* = 0.041), and Siamese cats (*p* = 0.003) in the referral population. The details of the proportions of breeds is provided in the Supplementary Materials (Table S4). When the control populations from the two referral centres were compared, there were 20 pure breeds and domestic cats that had statistically significantly different proportions. There were 12 breeds with higher proportions at MVSC and eight at UVTHS, with domestic cats overrepresented at UVTHS. The proportions of cases with significant differences are included in the Supplementary Materials (Table S5).

When the ages of the cats within the breeds were analysed, the cats with lymphoma were consistently older than the control cats of that breed. The weights of the cats with lymphoma within the breeds generally were lighter than the control cats of that breed. The exceptions were the British shorthair and Tonkinese breeds where the cats with lymphoma were heavier than the controls. This information is illustrated in the Supplementary Materials, ages are illustrated in Figure S1, and the weights in Figure S2.

When the anatomical presentation was reviewed for association with retrovirus status, 63 (88.7%) cats with gastrointestinal lymphoma tested negative for FIV compared to 221 (79.4%) overall (*p* < 0.05). There were no correlations with a positive status identified. The only anatomical site where a sex effect was found was for renal lymphoma, with males being over-represented (*p* = 0.002). There were 115 cases in males, compared to 55 in females. The anatomical sites of lymphoma within the breeds were compared and the sites that had a higher proportion of cases are listed in Table 3. Lymphoma cases by breed and site are included in Supplementary Materials (Table S2).

## 4. Discussion

This study provides a description of the demographics and clinical presentations of cats with lymphoma in Australia, presenting new information on breed risk of disease and relationships between breed and sex with anatomical presentation. Identification of eight breeds at increased risk of lymphoma and three at decreased risk, adds new information to that already reported. A notable feature identified within this study with large subsets of cases and controls, was that there were marked differences identified in breed risk based on the populations explored. For studies designed to evaluate lymphoma risk using methods such as genetic or microRNA patterns, there is a need to consider the use of broad data collection. Such desirable large case series can be achieved through the Australian and UK VetCompass initiatives, and central cancer databases such as the Australian Companion Animal Registry of Cancers. The two referral centres had been assumed to have similar populations, but a comparison of the breed frequencies between these two centres separated by 700 km identified 20 breeds in addition to domestic cats that were differentially represented in the hospital control populations. The difference between the centres may in part be due to the inclusion of cats seen by primary care at UVTHS in both the lymphoma and control groups, while MVSC had only referral cases. Approximately 30% of the cases at UVTHS were seen through primary care.

Seven breeds that were not previously identified as being at increased risk of lymphoma included the Abyssinian, Maine coon, Norwegian forest cat, ocicat, Persian, Russian blue, and Singapura. This presented study agrees with the findings of previous studies of an increased risk in Siamese cats [5,7,8,9]. However, our findings differ from previous results for two of these breeds in a European study that included 2868 cats with lymphoma, Maine coon and Norwegian forest cat, where they were reported to be at decreased risk [4]. When only referral populations were examined, the Turkish van was found to be at increased risk, contrary to the overall population where it was at decreased risk. The Birman, ragdoll, and Turkish van have not been previously reported to be at decreased risk. The Persian has previously been reported to be at decreased risk of lymphoma [4], a finding contrary to this study. The identified variation in proportions of breeds between the general and referral populations has the potential to introduce a bias into the breed risk calculations when select data sources are used. This adds support for using large population-based studies with well-aligned cases and controls. There was no recognisable pattern of the effect of breed proportions on breed risk. Abyssinian, chinchilla, and Devon rex cats were all over-represented in the referral control population with the Abyssinian identified as an increased risk in that population, while the other two were identified as being at increased risk in the general population.

The differences in breed risks identified in this study might reflect variations in breed popularity in different geographical areas, the relatively small numbers in less common breeds, differences in retroviral infection rates, vaccination rates, or genetic differences. Australia is reported to have a higher incidence of FIV and a lower incidence of FeLV than Switzerland [10,11], though the incidence of FeLV in Switzerland is falling [11]. Genetic differences are supported by the differences in blood groups in cats in differing geographical locations [12] as well as the potential for genetic bottlenecks that arise due to the strict quarantine regulations in Australia, restricting the importation of animals and biological products.

The significant reduction in risk for purebred-cross cats to both purebreds, and the population, has not been previously reported. There were insufficient numbers of pure breeds and their respective crosses to allow analysis within the breeds. While this might reflect a tendency for crossbred cats to be listed as domestic at some sites, leading to them being variably underreported in the populations, this potentially indicates an underlying genetic susceptibility within several breeds which is diminished in outbred populations. The insufficient cases of specific breed crosses to analyse in a meaningful manner and grouping all breed crosses together would include breeds with both an increased and decreased risk of lymphoma.

A male predisposition for lymphoma in cats has been reported previously [1,4,13], though other studies have not shown a sex difference [5,14]. Neutering has been reported as a risk factor in some studies [4,9], not significant in one [5], while only one had intact cats at increased risk [1]. This could not be assessed in this study due to the discrepancies noted between groups.

Retroviruses have an important role in the development of lymphoma in cats, with FeLV inducing mutagenesis [15,16], while FIV is facilitative [17]. Other viruses, such as feline gammaherpesvirus, might have a role in lymphomagenesis in cats [15,18]. Risk factors for both viruses include older age [19,20], being a sexually intact male [19,21] and outdoor access [19,20]. One small study from Australia showed approximately 50% of cats with lymphoma had FIV [7]. The prevalence of FeLV in cats with lymphoma is confounded by a large percentage of cats that have pro-viral DNA in tumour tissue but are antigen test negative [22,23,24]. There is variation in the accuracy of point-of-care test kits, the most common test performed in this study [25]. Details of retroviral status were not available in this study for the control population and most of the lymphoma cases, limiting interpretation.

The lymphoma cases in this study were statistically significantly older than the control populations, with about four years difference in median ages. This is in line with the reports that in the post-FeLV era, after the introduction of FeLV vaccinations and a decline in cases in endemic areas, lymphoma is considered a disease of older cats [5]. The age of the control populations is likely affected by the inclusion of kittens presented for their initial checks, vaccinations, and neutering in the UVTHS data. Councils require registration before six months of age, with registration that can be life-long, that would bias these data to younger cats.

The anatomical presentations of lymphoma identified in this study do not appear different from other reports in their distribution. Accurate comparison is difficult as there are inconsistencies in the classifications used in different studies [3,6,26]. The association between Siamese cats and mediastinal disease reported previously was not identified here [8,27], though the previously reported association with nasal disease was identified. There were differing distributions of lymphoma between the breeds, which while statistically significant might be influenced by low numbers of cases for some of these breeds. Larger studies would reduce the risk of Type 1 and Type 2 errors (false positive and false negative results).

There are several limitations in this study because of its retrospective nature and the sources of information used to generate the data. This includes some of the less common breeds with small numbers in both the lymphoma and reference populations, where a small error in classification could lead to a change in the significance of the findings. The lack of data on retroviral status for most lymphoma cases and all the control cases limits our ability to evaluate their role in lymphoma risk and the influence on anatomical presentations. The anatomical presentations might have been erroneous as the full history, examination, and imaging findings were not always available and the extent of staging was inconsistent.

The inclusion of multiple data sources was used to generate broader patient and reference populations; however, it reduced the ability to rule out that patients in the control population did not have lymphoma or develop lymphoma later, though the size of the population would limit any effect. Ideally, full records over a lifetime for the control and case populations would be used, which were not available to the authors. The choice of council registrations as part of the reference population was used as a random sample of the general population as only a limited number of councils responded to requests for feline data. With the requirement for microchipping of pet cats in many areas of Australia, would now allow these data, if available, might be used as a better comparison.

There might have been duplication of both cases and controls. In the referral hospital data, case identifiers were used to remove lymphoma cases from the control population. This was not possible for other sites and did not eliminate that lymphoma might have developed later. A review of all cases looking at breed, location based on postcode, and date of birth, revealed 11 cases (0.6% of lymphoma patients) where duplication was possible.

There was a discrepancy between the dates of data collection. The referral centre populations were taken from a 20-year period, the reference laboratory from a 2.33-year period, the corporate practice from a 3-year period and the council registration data from a single year. Breed preferences could have changed over that period, and this might have influenced the results. Inaccuracies in entering breed data, especially for owner inputted data such as the council registration, is another potential source of error. In Australia there has not been a major change in retroviral status during this period [10].

## 5. Conclusions

This study will not have an immediate impact on clinical practice in Australia but provides a reference on which other studies exploring the aetiology of lymphoma in cats can be based. Additional studies with a large group of cases and reference populations drawn from the same source are required to confirm the findings of this study. The differences in breed risk can facilitate the identification of markers of disease risk, which are needed in this species given the paucity of genetic or other studies currently available. With increased information, advice to breeders and owners of cats can be developed with an aim to reduce the prevalence of this disease. Study results may help to guide research studies, particularly in considering potential differences based on the sources used for epidemiological studies.

## Figures and Tables

**Table 1:** Lymphoma locations, grade, type and retroviral status.

Parameter	Number	Percentage
Location		
Gastrointestinal	671	39.4
Multicentric	422	24.6
Renal	171	10.0
Nasal	167	9.8
Hepatic	66	3.9
Mediastinal	55	3.2
Cutaneous	44	2.8
Central nervous system	19	1.1
Other	60	3.5
Unknown	30	1.9
Grade (*n* = 1510)		
High	1173	77.7
Intermediate	95	6.3
Low	241	16.0
Indolent	1	
Immunophenotype or other typing (*n* = 102)		
B-cell	37	36.3
T-cell	24	23.5
Non-B-non-T	1	1.0
T-zone	1	1.0
T-cell-rich-B-cell	4	4.0
Epitheliotropic (untyped)	1	1.0
Large-granular	34	33.3
Retrovirus status in cats with results available		
FeLV positive	13	4.6
FIV positive	65	20.6

**Table 2:** Odds ratios for breeds identified as being at increased or decreased risk of lymphoma.

Breed	N=	Odds Ratio	95% Confidence Intervals	*p* Value
Breeds at increased risk				
Abyssinian	19	2.1	1.4–3.4	0.001
Maine coon	13	2.2	1.2–3.8	0.006
Norwegian forest cat	5	3.1	1.3–7.7	0.014
Ocicat	4	6.5	2.3–18.4	<0.001
Persian	23	1.9	1.2–2.8	0.004
Russian blue	46	2.0	1.5–2.7	<0.001
Siamese	68	2.6	2.0–3.3	<0.001
Singapura	3	6.0	1.8–20.0	0.003
Breeds at decreased risk				
Birman	13	0.5	0.3–0.9	0.031
Ragdoll	9	0.1	0.1–0.3	<0.001
Turkish van	3	0.3	0.1–0.9	0.028
Purebred cross	18	0.4	0.2–0.6	<0.001

**Table 3:** List of breeds that had lymphoma occur proportionately more in some anatomical sites than others.

Breed	Site of Higher Proportion	Compared to Site	*p* Value
Australian mist	CNS	Multicentric	0.014
Ragdoll	CNS	Multicentric	0.014
British shorthair	CNS	Gastrointestinal	0.016
British shorthair	CNS	Multicentric	0.004
Burmilla	Nasal	Gastrointestinal	0.006
Siamese	Nasal	Gastrointestinal	0.001
Siamese	Nasal	Multicentric	0.002
Domestic	Cutaneous	Nasal	0.0012
Domestic	Hepatic	Nasal	<0.001
Domestic	Multicentric	Nasal	0.001
Domestic	Renal	Nasal	0.029
Domestic	Hepatic	CNS	0.002
Domestic	Hepatic	Gastrointestinal	0.011

## Data Availability

The data for this study is included in the Supplementary Materials and was part of a Ph.D. thesis by the first author, available from the University of Sydney thesis repository.

## Supplementary Materials

The following supporting information can be downloaded at: https://www.mdpi.com/article/10.3390/vetsci11120641/s1, Figure S1: Ages of cats by breed comparing the control cats to lymphoma cases; Figure S2: Weights of cats by breed comparing the control cats to lymphoma cases; Table S1: List of breeds with numbers of control and lymphoma cases including the site from where they were derived; Table S2: List of the number of lymphoma cases listed by anatomical site and breed; Table S3: Odds ratios, 95% CI, and *p* values for lymphoma risk in all breeds that had lymphoma cases; Table S4: A comparison of the breed proportions between the referral and non-referral sources for control and lymphoma cases when a significant difference was found; Table S5: A comparison of the breed proportions between the control populations of the two referral centres when a significant difference was found.

## References

[1] Schneider R. (1983). Comparison of Age- and Sex-Specific Incidence Rate Patterns of the Leukemia Complex in the Cat and the Dog. JNCI J. Natl. Cancer Inst..

[2] Bartlett P.C., Van Buren J.W., Neterer M., Zhou C. (2010). Disease surveillance and referral bias in the veterinary medical database. Prev. Vet. Med..

[3] Mason S., Pittaway C. (2022). Feline lymphoma: Diagnosis, staging and clinical presentations. Practice.

[4] Graf R., Gruentzig K., Boo G., Haessig M., Axhausen K.W., Fabrikant S., Walle M., Meier D., Guscetti F., Folkers G. (2016). Swiss Feline Cancer Registry 1965–2008: The Influence of Sex, Breed and Age on Tumour Types and Tumour Locations. J. Comp. Pathol..

[5] Louwerens M., London C.A., Pedersen N.C., Lyons L.A. (2005). Feline lymphoma in the post-feline leukemia virus era. J. Vet. Intern. Med..

[6] Economu L., Stell A., O’Neill D.G., Schofield I., Stevens K., Brodbelt D. (2021). Incidence and risk factors for feline lymphoma in UK primary-care practice. J. Small Anim. Pract..

[7] Court E.A., Watson A.D.J., Peaston A.E. (1997). Retrospective study of 60 cases of feline lymphosarcoma. Aust. Vet. J..

[8] Gabor L.J., Malik R., Canfield P.J. (1998). Clinical and anatomical features of lymphosarcoma in 118 cats. Aust. Vet. J..

[9] Rissetto K., Villamil J.A., Selting K.A., Tyler J., Henry C.J. (2011). Recent Trends in Feline Intestinal Neoplasia: An Epidemiologic Study of 1,129 Cases in the Veterinary Medical Database from 1964 to 2004. J. Am. Anim. Hosp. Assoc..

[10] Westman M.E., Paul A., Malik R., McDonagh P., Ward M.P., Hall E., Norris J.M. (2016). Seroprevalence of feline immunodeficiency virus and feline leukaemia virus in Australia: Risk factors for infection and geographical influences (2011–2013). J. Feline Med. Surg. Open Rep..

[11] Hofmann-Lehmann R., Gonczil E., Riond B., Meli M.L., Willi B., Howard J., Schaarschmidt D., Regli W., Gilli U., Boretti F.S. (2018). Feline leukemia virus infection: Importance and current situation in Switzerland. Schweiz. Arch. Tierheilkd..

[12] Knottenbelt C. (2002). The Feline AB Blood Group System and its Importance in Transfusion Medicine. J. Feline Med. Surg..

[13] Loar A.S. (1984). The management of feline lymphosarcoma. Vet. Clin. N. Am.-Small Anim. Pract..

[14] Crighton G.W. (1969). Feline leukaemia (Lymphosarcoma) symposium. 1. Diagnosis of leukaemia in the cat. J. Small Anim. Pract..

[15] Beatty J.A., Troyer R.M., Carver S., Barrs V.R., Espinasse F., Conradi O., Stutzman-Rodriguez K., Chan C.C., Tasker S., Lappin M.R. (2014). Felis catus gammaherpesvirus 1; a widely endemic potential pathogen of domestic cats. Virology.

[16] Fujino Y., Ohno K., Tsujimoto H. (2008). Molecular pathogenesis of feline leukemia virus-induced malignancies: Insertional mutagenesis. Vet. Immunol. Immunopathol..

[17] Beatty J. (2014). Viral causes of feline lymphoma: Retroviruses and beyond. Vet. J..

[18] McLuckie A.J., Barrs V.R., Lindsay S., Aghazadeh M., Sangster C., Beatty J.A. (2018). Molecular Diagnosis of Felis catus Gammaherpesvirus 1 (FcaGHV1) Infection in Cats of Known Retrovirus Status with and without Lymphoma. Viruses.

[19] Burling A.N., Levy J.K., Scott H.M., Crandall M.M., Tucker S.J., Wood E.G., Foster J.D. (2017). Seroprevalences of feline leukemia virus and feline immunodeficiency virus infection in cats in the United States and Canada and risk factors for seropositivity. JAVMA-J. Am. Vet. Med. Assoc..

[20] Barros V.R., Bezerra J.A.B., Bochnakian M.S., de Paula V.V., Filgueira K.D. (2017). Epidemiology of feline immunodeficiency virus and feline leukemia virus in a veterinary teaching hospital. Braz. J. Hyg. Anim. Sanity.

[21] da Costa F.V.A., Valle S.D., Machado G., Corbellini L.G., Coelho E.M., Rosa R.B., Gonzalez F.H.D. (2017). Hematological findings and factors associated with feline leukemia virus (FeLV) and feline immunodeficiency virus (FIV) positivity in cats from southern Brazil. Pesqui. Vet. Bras..

[22] Gabor L.J., Jackson M.L., Trask B., Malik R., Canfield P.J. (2001). Feline leukaemia virus status of Australian cats with lymphosarcoma. Aust. Vet. J..

[23] Hartmann K. (2011). Clinical aspects of feline immunodeficiency and feline leukemia virus infection. Vet. Immunol. Immunopathol..

[24] Weiss A.T.A., Klopfleisch R., Gruber A.D. (2010). Prevalence of feline leukaemia provirus DNA in feline lymphomas. J. Feline Med. Surg..

[25] Westman M.E., Malik R., Hall E., Sheehy P.A., Norris J.M. (2017). Comparison of three feline leukaemia virus (FeLV) point-of-care antigen test kits using blood and saliva. Comp. Immunol. Microbiol. Infect. Dis..

[26] Versteegh H., Zandvliet M.M., Feenstra L.R., van der Steen F.E., Teske E. (2023). Feline Lymphoma: Patient Characteristics and Response Outcome of the COP-Protocol in Cats with Malignant Lymphoma in The Netherlands. Animals.

[27] Fabrizio F., Calam A.E., Dobson J.M., Middleton S.A., Murphy S., Taylor S.S., Schwartz A., Stell A.J. (2014). Feline mediastinal lymphoma: A retrospective study of signalment, retroviral status, response to chemotherapy and prognostic indicators. J. Feline Med. Surg..

