# Supplementary material for: Demographics of Feline Lymphoma in Australian Cat Populations: 1705 Cases
Source: Vet Sci. 2024 Dec 11;11(12):641. doi: 10.3390/vetsci11120641 (PMC11680128; doi:10.3390/vetsci11120641)
Supplement: Supplementary file 1 [file vetsci-11-00641-s001.zip › vetsci-3210888-supplementary.pdf]

Supplementary Materials

Figure S1: Ages of cats by breed comparing the control cats to lymphoma cases. Significant differences (p<0.05) are indicated by an * after the breed's name.

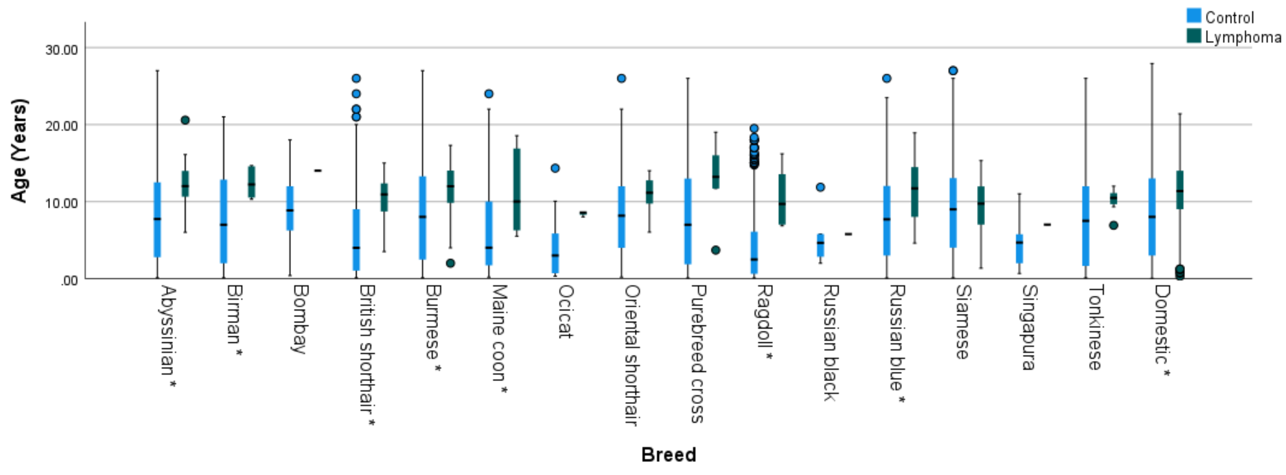

Figure S2: Weights of cats by breed comparing the control cats to lymphoma cases. Significant differences (p<0.05) are indicated by an * after the breed's name.

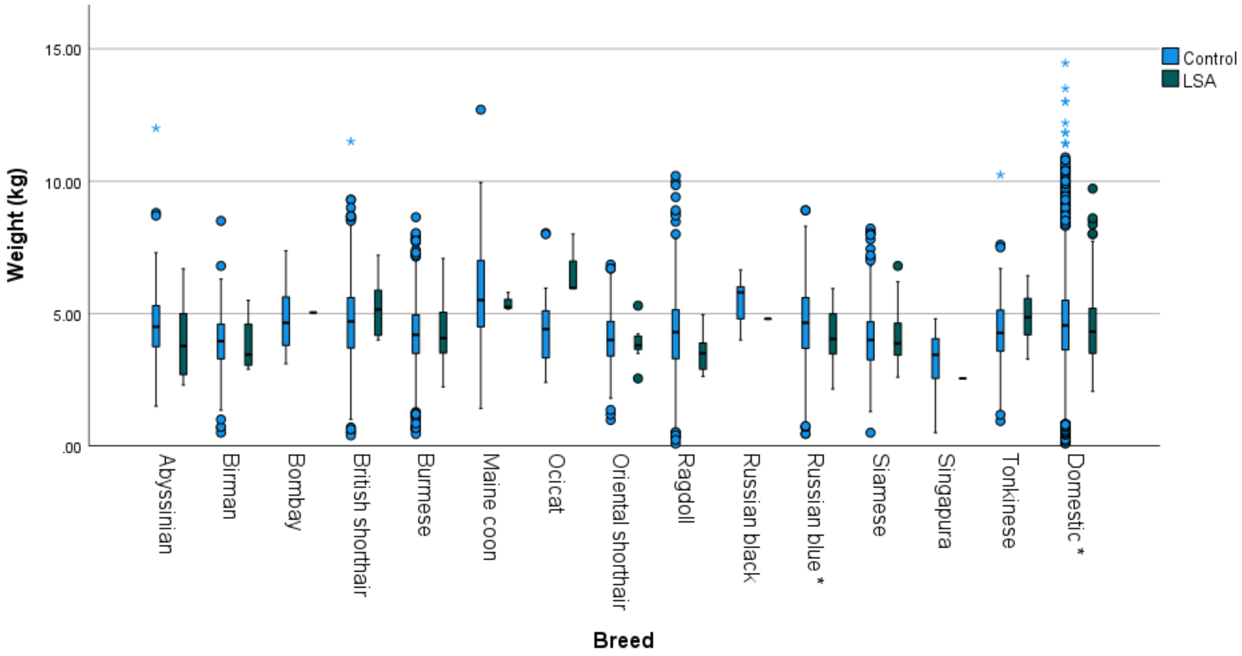

Table S1: List of breeds with numbers of control and lymphoma cases including the site from where they were derived.

	Controls		Lymphoma cases			
Breed	Council	Referral Centres	GX	Idexx	Non-referral Total	Referral Centres
Abyssinian	171	277	1	4	5	14
American shorthair	1	26	0	0	0	0
Australian Mist	165	139	2	1	3	6
Australian Tiffany	86	18	0	0	0	1
Balinese	39	32	0	2	2	1
Bengal	308	285	0	7	7	7
Birman	739	451	2	7	9	4
Bombay	39	21	0	0	0	1
British shorthair	779	718	0	11	11	10
Burmese	3097	1955	6	48	54	40
Burmilla	283	85	0	0	0	4
Chartreux	12	3	0	0	0	0
Chinchilla	66	145	1	4	5	1
Cornish rex	103	140	1	2	3	2
Cymric	2	0	0	0	0	0
Devon Rex	340	399	2	9	11	7
Domestic	39999	21655	121	585	706	541
Egyptian Mau	9	10	0	0	0	0
European shorthair	28	43	0	0	0	0
Exotic shorthair	60	148	0	0	0	1
Havana	177	5	0	0	0	0
Highland	60	2	0	0	0	0
Himalayan	173	259	1	3	4	5
Japanese bobtail	9	0	0	0	0	0
Javanese	2	0	0	0	0	0
Korat	15	28	0	2	2	0
La Perm	4	0	0	0	0	0
Lykoi	80	2	0	0	0	0
Maine Coon	117	184	1	8	9	4
Manx	54	43	2	0	2	0
Munchkin	14	18	0	0	0	0
Nebelung	17	2	0	0	0	0
Norwegian forest cat	21	60	1	2	3	2

Table S1 (Continued): List of breeds with numbers of control and lymphoma cases including the site from where they were derived.

	Controls		Lymphoma cases			
**Breed**	**Council**	**Referral Centres**	**GX**	**Idexx**	**Non-referral total**	**Referral Centres**
Ocicat	5	26	0	1	1	3
Oriental longhair	185	7	0	0	0	0
Oriental shorthair	515	162	0	5	5	10
Persian	15	614	1	9	10	13
Pixie Bob	7	0	0	0	0	0
Purebred cross	1899	484	6	12	18	0
Ragdoll	1922	1229	0	4	4	5
Russian black	0	5	0	0	0	1
Russian blue	657	533	3	16	19	27
Scottish fold	74	127	0	2	2	2
Scottish shorthair	39	0	0	0	0	0
Selkirk rex	41	20	0	0	0	0
Siamese	679	663	5	20	25	43
Siberian	41	26	0	0	0	1
Singapura	6	19	0	2	2	1
Snowshoe	9	23	0	0	0	1
Somali	33	56	0	1	1	0
Sphynx	32	75	0	0	0	0
Tonkinese	384	265	0	13	13	7
Turkish angora	9	21	0	0	0	1
Turkish van	40	35	0	0	0	3
Unknown	537	0	0	0	0	0
	**54198**	**31543**	**156**	**780**	**936**	**769**

Table S2: List of number of lymphoma case listed by anatomical site and breed.

Breed	Unknown	CNS	Cutaneous	GI	Hepatic	Mediastinal	Multicentric	Nasal	Other	Renal	Total
Abyssinian	0	0	0	13	1	0	2	3	0	0	**19**
Australian mist	0	1	0	4	0	0	1	2	1	0	**9**
Australian Tiffany	0	0	0	1	0	0	0	0	0	0	**1**
Balinese	0	0	0	3	0	0	0	0	0	0	**3**
Bengal	1	0	0	6	0	0	4	2	0	1	**14**
Birman	1	0	0	7	0	1	2	1	1	0	**13**
Bombay	0	0	0	0	0	0	0	0	0	1	**1**
British shorthair	0	2	0	9	0	0	4	2	0	4	**21**
Burmese	2	1	1	35	2	1	31	13	3	5	**94**
Burmilla	0	0	0	1	0	0	0	3	0	0	**4**
Chinchilla	0	0	0	3	0	0	2	1	0	0	**6**
Cornish rex	0	0	0	0	0	0	1	3	1	0	**5**
Devon rex	1	1	1	6	0	0	7	1	1	0	**18**
Domestic	20	10	39	480	61	45	323	99	39	131	**1247**
Exotic shorthair	0	0	0	0	0	0	0	0	1	0	**1**
Himalayan	0	0	0	3	0	0	3	2	0	1	**9**
Korat	0	0	0	2	0	0	0	0	0	0	**2**
Maine coon	1	0	0	8	0	0	1	2	1	0	**13**
Manx	0	0	0	0	0	0	1	0	1	0	**2**
Norwegian forest	0	0	0	2	0	0	2	0	0	1	**5**
Ocicat	1	0	0	0	0	0	0	0	2	1	**4**
Oriental shorthair	1	1	0	5	0	0	3	3	1	1	**15**
Persian	0	0	1	12	1	0	2	3	0	4	**23**
Purebred cross	2	0	0	5	0	1	4	2	1	3	**18**
Ragdoll	0	1	0	5	0	1	1	0	0	1	**9**
Russian black	0	0	0	0	0	0	0	0	0	1	**1**
Russian blue	0	0	1	24	0	1	9	2	2	7	**46**
Scottish fold	0	0	0	1	0	0	2	1	0	0	**4**
Siamese	0	1	0	21	1	5	12	18	4	6	**68**
Siberian	0	0	0	1	0	0	0	0	0	0	**1**
Singapura	0	0	0	2	0	0	1	0	0	0	**3**
Snowshoe	0	1	0	0	0	0	0	0	0	0	**1**
Somali	0	0	0	1	0	0	0	0	0	0	**1**
Tonkinese	0	0	1	9	0	0	4	3	1	2	**20**
Turkish angora	0	0	0	0	0	0	0	0	0	1	**1**
Turkish van	0	0	0	2	0	0	0	1	0	0	**3**
**Total**	**30**	**19**	**44**	**671**	**66**	**55**	**422**	**167**	**60**	**171**	**1705**

Table S3: Odds ratios, 95% CI, and p values for lymphoma risk in all breeds that had lymphoma cases. Significant values are highlighted in bold.

Breed	All cases			Cases from referral centres			Cases from non-referral sources		
	OR	95% CI	p value	OR	95% CI	p value	OR	95% CI	p value
Abyssinian	**2.1**	**1.4-3.4**	**0.001**	**2.1**	**1.2-3.6**	**0.008**	1.6	0.7-3.9	0.3
Australian mist	1.5	0.8-2.9	0.239	1.8	0.8-4.0	0.17	1.1	0.3-3.3	0.93
Australian Tiffany	0.5	0.1-3.4	0.468	2.3	0.3-17.1	0.423			
Balinese	2.1	0.7-6.8	0.201	1.3	0.2-9.4	0.807	3.0	0.7-12.3	0.133
Bengal	1.2	0.7-2.0	0.524	1.0	0.5-2.1	0.984	1.3	0.6-2.8	0.471
Birman	**0.5**	**0.3-0.9**	**0.031**	**0.4**	**0.1-1.0**	**0.043**	0.7	0.4-1.4	0.294
Bombay	0.8	0.1-6.0	0.861	1.3	0.2-9.4	0.807			
British shorthair	0.7	0.5-1.1	0.109	0.6	0.3-1.1	0.075	0.8	0.4-1.5	0.504
Burmese	0.9	0.8-1.1	0.51	0.8	0.6-1.1	0.257	1.0	0.8-1.3	0.943
Burmilla	0.5	0.2-1.5	0.228	1.9	0.7-5.3	0.198			
Chinchilla	1.4	0.6-3.2	0.387	0.3	0.0-2.0	0.207	**4.4**	**1.8-11.0**	**0.001**
Cornish rex	1.0	0.4-2.5	0.94	0.6	0.1-2.4	0.452	1.7	0.5-5.3	0.372
Devon rex	1.2	0.8-2.0	0.393	0.7	0.3-1.5	0.385	**1.9**	**1.0-3.4**	**0.04**
Domestic	1.1	1.0-1.2	0.263	1.1	0.9-1.3	0.316	1.1	0.9-1.3	0.262
Exotic shorthair	0.2	0.0-1.7	0.156	0.3	0.0-2.0	0.2			
Himalayan	1.0	0.5-2.0	0.89	0.8	0.3-1.9	0.604	**15.5**	**5.1-46.8**	**<0.0001**
Korat	2.3	0.6-9.7	0.24				**7.7**	**1.8-33.9**	**0.007**
Maine coon	**2.2**	**1.2-3.8**	**0.006**	0.9	0.3-2.4	0.82	**4.5**	**2.3-8.9**	**<0.0001**
Manx	1.0	0.3-4.2	0.96				2.1	0.5-8.8	0.289
Norwegian forest cat	**3.1**	**1.3-7.7**	**0.014**	1.4	0.3-5.6	0.663	**8.3**	**2.5-27.9**	**0.001**
Ocicat	**6.5**	**2.3-18.4**	**<0.0001**	**4.7**	**1.4-15.7**	**0.011**	**11.6**	**1.4-99.3**	**0.025**
Oriental shorthair	1.1	0.7-1.9	0.677	**2.6**	**1.3-4.9**	**0.004**	0.6	0.2-1.4	0.198
Persian	**1.9**	**1.2-2.8**	**0.004**	0.9	0.5-1.5	0.6	**39.0**	**17.5-87.0**	**<0.0001**
Purebred cross	**0.4**	**0.2-0.6**	**<0.0001**				**0.5**	**0.3-0.9**	**0.01**
Ragdoll	**0.1**	**0.1-0.3**	**<0.0001**	**0.2**	**0.1-0.4**	**<0.0001**	**0.1**	**0.0-0.3**	**<0.0001**
Russian black	**10.1**	**1.2-86.2**	**0.035**	8.2	1.0-70.4	0.055			
Russian blue	**2.0**	**1.5-2.7**	**<0.0001**	**2.1**	**1.4-3.1**	**<0.0001**	**1.7**	**1.1-2.7**	**0.026**
Scottish fold	1.0	0.4-2.7	0.999	0.6	0.2-2.3	0.539	1.6	0.4-6.4	0.532
Siamese	**2.6**	**2.0-3.3**	**<0.0001**	**2.8**	**2.0-3.8**	**<0.0001**	**2.2**	**1.4-3.2**	**<0.0001**
Siberian	0.8	0.1-5.4	0.776	1.6	0.2-11.6	0.654			
Singapura	**6.0**	**1.8-20.0**	**0.003**	2.2	0.3-16.2	0.453	**19.3**	**3.9-95.9**	**<0.0001**
Snowshoe	1.6	0.2-11.5	0.656	1.8	0.2-13.2	0.571			
Somali	0.6	0.1-4.0	0.569				1.8	0.2-12.8	0.58
Tonkinese	1.6	1.0-2.4	0.053	1.1	0.5-2.3	0.833	**2.0**	**1.1-3.4**	**0.017**
Turkish angora	1.7	0.2-12.3	0.061	2.0	0.3-14.5	0.513			
Turkish van	**0.3**	**0.1-0.9**	**0.028**	**3.5**	**1.1-11.5**	**0.004**			

Table S4: A comparison of the breed proportions between the referral and non-referral sources for control and lymphoma cases when a significant difference was found. The proportion of controls or cases for each breed for each source and the p value are listed, with the over-represented group highlighted in bold.

	Controls			Lymphoma cases		
**Breed**	**Referral Proportion**	**Non-referral Proportion**	**p value**	**Referral proportion**	**Non-referral proportion**	**p value**
Abyssinian	**0.88%**	**0.32%**	**<0.001**	**1.82%**	0.53%	**0.018**
American shorthair	**0.08%**	<0.01%	**<0.001**			
Australian Mist	**0.44%**	0.3%	**0.002**			
Australian Tiffany	0.06%	**0.16%**	**<0.001**			
Bengal	**0.9%**	0.57%	**<0.001**			
British shorthair	**2.28%**	1.44%	**<0.001**			
Burmese	**6.2%**	5.71%	**0.004**			
Burmilla	0.27%	**0.52%**	**<0.001**	**0.52%**	0.0%	**0.041**
Chinchilla	**0.46%**	0.12%	**<0.001**			
Cornish rex	**0.44%**	0.19%	**<0.001**			
Devon Rex	**1.26%**	0.63%	**<0.001**			
Domestic	68.65%	**73.8%**	**<0.001**	70.35%	**75.43%**	**0.021**
European shorthair	**0.14%**	0.05%	**<0.001**			
Exotic shorthair	**0.47%**	0.11%	**<0.001**			
Himalayan	**0.82%**	0.32%	**<0.001**			
Japanese bobtail	0.0%	**0.02%**	**0.031**			
Korat	**0.09%**	0.03%	**<0.001**			
Lykoi	0.01%	**0.15%**	**<0.001**			
Maine Coon	**0.58%**	0.22%	**<0.001**			
Nebelung	0.01%	**0.03%**	**0.017**			
Norwegian forest cat	**0.19%**	0.04%	**<0.001**			
Ocicat	**0.08%**	0.01%	**<0.001**			
Oriental longhair	0.02%	**0.34%**	**<0.001**			
Oriental shorthair	0.51%	**0.95%**	**<0.001**			
Persian	**1.95%**	0.03%	**<0.001**			
Purebred cross	1.53%	**3.5%**	**<0.001**	0.0%	**1.92%**	**<0.001**
Ragdoll	**3.9%**	3.55%	**0.009**			
Russian black	**0.02%**	0.0%	**0.007**			
Russian blue	**1.69%**	1.21%	**<0.001**			
Scottish fold	**0.4%**	0.14%	**<0.001**			
Scottish shorthair	0.0%	**0.07%**	**<0.001**			
Siamese	**2.1%**	1.25%	**<0.001**	**5.59%**	2.67%	**0.003**
Singapura	**0.06%**	0.01%	**<0.001**			
Snowshoe	0.07%	**0.17%**	**0.037**			
Somali	**0.18%**	0.06%	**<0.001**			
Sphynx	**0.24%**	0.06%	**<0.001**			
Tonkinese	**0.84%**	0.71%	**0.034**			
Turkish angora	**0.07%**	0.02%	**<0.001**			
Unknown	0.0%	**0.99%**	**<0.001**			

Table S5: A comparison of the breed proportions between the control populations of the two referral centres when a significant difference was found. The proportion of controls for each breed for each site and the p value are listed, with the over-represented group highlighted in bold.

	**Controls**		
**Breed**	**MVSC**	**UVTHS**	**p value**
Abyssinian	**1.42%**	0.68%	**<0.001**
Australian Tiffany	0.0%	**0.08%**	**0.01**
Balinese	**0.17%**	0.08%	**0.032**
Birman	**2.07%**	1.19%	**<0.001**
Chinchilla	**0.82%**	0.33%	**<0.001**
Devon Rex	**1.59%**	1.15%	**0.002**
Domestic	66.54%	**69.42%**	**<0.001**
European shorthair	0.0%	**0.19%**	**<0.001**
Oriental shorthair	**0.70%**	0.45%	**0.005**
Persian	**2.63%**	1.70%	**<0.001**
Purebred cross	0.52%	**1.90%**	**<0.001**
Ragdoll	2.84%	**4.28%**	**<0.001**
Russian black	**0.047%**	0.004%	**0.007**
Russian blue	**1.95%**	1.59%	**0.028**
Selkirk rex	0.012%	**0.082%**	**0.028**
Siamese	**3.42%**	1.62%	**<0.001**
Siberian	0.012%	**0.108%**	**0.008**
Singapura	**0.118%**	0.039%	**0.011**
Sphynx	0.14%	**0.27%**	**0.035**
Tonkinese	**1.05%**	0.76%	**0.012**
Turkish van	0.05%	**0.13%**	**0.040**
